# An Unexpected Hepatic Hydrothorax After a Successful Kasai Portoenterostomy: A Case Report

**DOI:** 10.3389/fped.2021.766187

**Published:** 2021-11-01

**Authors:** Giulia Ranucci, Fabiola Di Dato, Daniela Liccardo, Marco Spada, Giuseppe Maggiore, Raffaele Iorio

**Affiliations:** ^1^Section of Pediatrics, Department of Translational Medical Science, University of Naples Federico II, Naples, Italy; ^2^Hepatology, Gastroenterology, Nutrition and Liver Transplant Unit, IRCCS Bambino Gesù Pediatric Hospital, Rome, Italy; ^3^Division of Hepatobiliopancreatic Surgery, Liver and Kidney Transplantation, IRCCS Bambino Gesù Pediatric Hospital, Rome, Italy

**Keywords:** ascites, biliary atresia, portal hypertension, liver transplant, liver cirrhosis

## Abstract

Hepatic hydrothorax (HH) represents a rare complication of portal hypertension among adult cirrhotic patients. Here, we describe a pediatric case of HH, observed in a biliary atresia infant. The child presented with recurrent right-sided pleural effusion, after a successful Kasai portoenterostomy with restoration of bile flow and without overt signs of hepatic failure. Recurrence of HH led the patient to liver transplant despite a low pediatric end-stage liver disease value. Although rare, HH can also occur in children and should be suspected in patients with portal hypertension and respiratory distress. HH may be an indication for liver transplantation.

## Introduction

Biliary atresia (BA) is a rare, idiopathic, progressive disease leading to death if untreated. Kasai portoenterostomy (KPE) is the initial treatment to restore bile flow in order to delay, or even prevent, the natural progression to cirrhosis. If KPE is not successful, liver transplant (LT) is usually required within a year due to complications of portal hypertension (PHT) or liver failure (1). If KPE is successful, a small group will survive long term with native liver, even if in about 70% of cases, despite restoring bile flow, PHT progresses with the need for LT over the years (2). Hepatic hydrothorax (HH) is defined as the accumulation of transudative pleural effusion occurring in 5–15% of patients with cirrhotic PHT in the absence of cardiopulmonary, renal, inflammatory, or malignant diseases, and it is associated with high mortality (3). The occurrence of HH is associated with hepatic dysfunction, but not directly with serum albumin levels (4). In most cases (85%), HH develops on the right side and can present without ascites in a small proportion of patients (5). Recently, this complication was reported in an infant with a severe form of BA judged not eligible for KPE (6). Instead, HH was never described in children successfully treated with Kasai intervention. Here, we describe a 7-month-old girl with BA presenting with recurrent HH occurring after a successful KPE with restoration of bile flow and without overt signs of hepatic failure.

## Case Description

**Figure 1:**
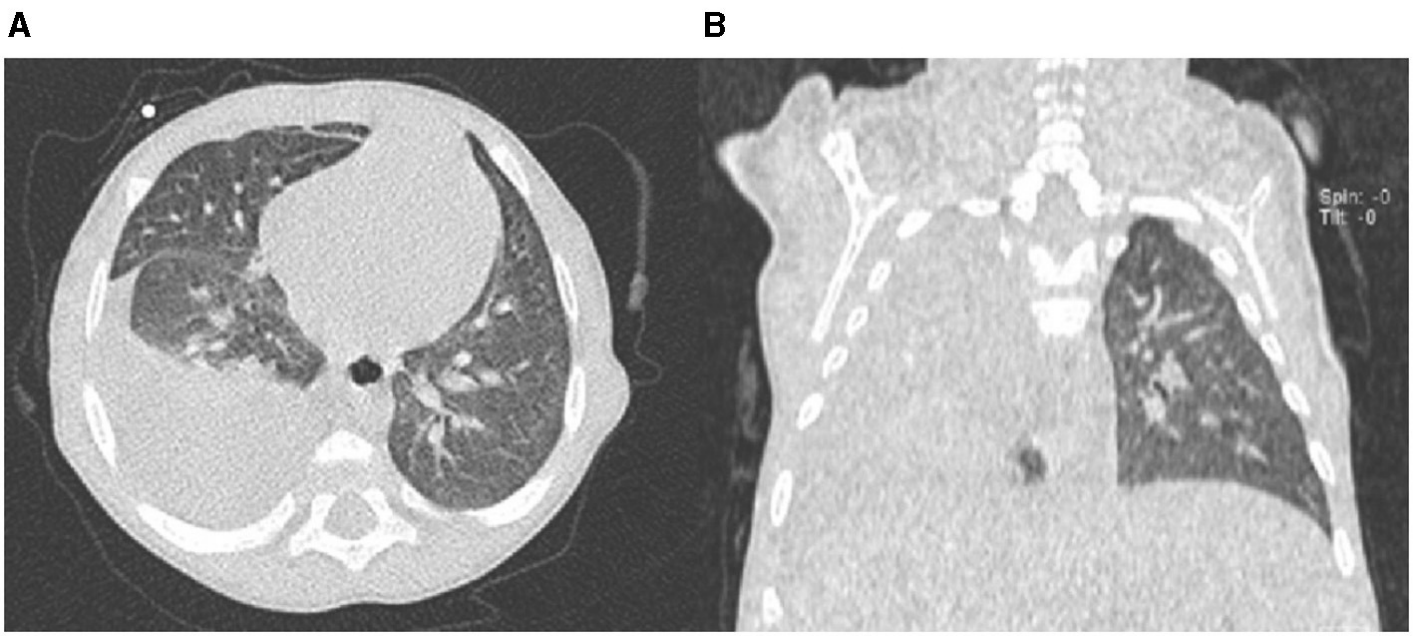
Chest computed tomography (CT) scan axial view **(A)** and coronal view **(B)** showing right-sided pleural effusion.

A 3-month-old girl was referred for jaundice, hepatomegaly, acholic stools, and dark urine. At blood testing, conjugated hyperbilirubinemia, thrombocytosis, and raised liver enzymes were found, and triangular cord sign was observed at liver ultrasound. Cholangiography confirmed BA, and KPE was performed at the age of 94 days. At surgical exploration, the liver was fully mobilized and appeared enlarged and greenish-brown in color with increased consistency and macronodular surface. Hepatic histological evaluation revealed severe fibrosis (METAVIR F3–F4). During the following 4 months, the girl was in good clinical conditions, and bile flow was completely restored within 1 month after KPE with total bilirubin serum levels falling under 1 mg/dl. Unfortunately, she developed refractory ascites requiring chronic diuretic treatment with furosemide (1 mg/kg/day) and spironolactone (3 mg/kg/day). At the age of 7 months, the patient experienced a sudden respiratory distress. On admission, she was apyretic with subcostal and jugular retractions. Thoracic auscultation revealed decreased airflow in the right basal region, with oxygen saturation at room air 94%. Laboratory data showed normal liver function tests (albumin 3.2 g/dl; prothrombin time international normalized ratio 1.2) with normal values of aspartate aminotransferase, alanine aminotransferase, and gamma-glutamyltransferase; conjugated serum bilirubin was 0.6 mg/dl. Blood count was normal (platelet count 230,000/μl), and C-reactive protein was negative. Echocardiogram revealed normal left ventricular wall motion and contractility. Chest X-ray and computed tomography (CT) scan showed pleural effusion with an almost complete opacification of the right hemithorax (Figure 1). Rapid deterioration of pulmonary function led to an emergency intubation, and thoracentesis drained about 250 ml of transudate (proteins = 1.3 g/dl, 1 leukocytes/mm^3^, sterile).

Subsequently, in combination with diuretic therapy, non-invasive ventilation (NIV) with full-face mask to high-flow nasal cannula oxygen (HNFC) was started. Infections (including tuberculosis) and inflammatory, renal, and neoplastic diseases were also excluded. Esophagogastroduodenoscopy showed absence of esophageal varices. After discharge, pleural effusion recurred three times despite aggressive diuretic therapy; in one circumstance, imaging revealed only minimal ascitic effusion. In the following months, serial ultrasound evaluations confirmed persistent perihepatic and perisplenic ascites with fluid between intestinal loops and showed signs of PHT with hepatofugal venous flow.

At 10 months of age, despite a low pediatric end-stage liver disease (PELD) score (equal to 3), the infant was listed for LT due to repeated episodes of respiratory distress, the need for multiple thoracentesis, and frequent hospitalizations. At the age of 11 months, the patient underwent deceased donor split LT. During surgery, no vascular, diaphragm, or azygous vein changes were identified. Intraoperatively, PHT was advanced, and the native liver appeared enlarged and with a multinodular cirrhotic aspect. Histological evaluation showed signs of biliary cirrhosis due to BA. In the following 12 months after transplantation, the infant showed good liver function and no further HH relapse.

## Discussion

At the best of our knowledge, this is the first pediatric case of HH observed in a BA infant after successful KPE with complete normalization of bilirubin levels. The monolaterality of pleural effusion, the absence of other etiologies, and the presence of liver cirrhosis suggested in our patient a hydrothorax of hepatic origin as described in adults. The pathophysiology of HH includes the passage of ascitic fluid through small diaphragmatic defects or the increased pressure and flow of the azygous vein leading to plasma leakage. Negative intrathoracic pressure facilitates the risk of fluid passage from peritoneal to pleural compartment (3). Although most cases of HH are associated with ascites, there are also patients with HH without ascites.

On the other hand, the presence of PHT is a constant in patients with HH (7). It remains unclarified why HH occurs only in a small subset of cirrhotic adults and almost never in children. A case of HH was recently reported in a cirrhotic infant with BA in whom PHT was so advanced as to preclude KPE at the age of 2 months (6). Our case instead focused attention on the unexpected occurrence of HH in a jaundice-free patient with normal liver function after a successful KPE. If the severity of fibrosis at the time of KPE could explain the development of early PHT in both the cases, it remains unexplained why PHT manifested itself so early with HH and not with bleeding esophageal varices. It is noteworthy that at the time of the LT neither the patient of Morin et al. nor ours presented diaphragmatic lesions explaining the passage of fluids from the abdomen to the pleura. In the patient of Morin et al., there was a macroscopic hole in the right diaphragm that was no longer present at the time of the LT. As for management of HH, Morin et al. stressed the role of an implantable pleural access device for controlling pleural effusion as a bridging solution to LT (6). On the other hand, our case, according with the current recommendations for adult patients, emphasized the therapeutic role of LT that can also be successfully performed in young infants (7). In our case, the choice not to carry out palliative surgical procedures was linked to the risk of hindering the success of the transplant procedure. In addition, it is well-known that chest tube placement is associated with a higher mortality rate and increased risk of hepatorenal syndrome and infections (8). A further message from this case report concerns the role of PELD in selecting candidates for LT. In spite of the severity of her clinical condition due to relapsing life-threatening HH episodes, our patient had a low PELD value, which could affect transplantation. As for adults, liver allocation in children should take into account not only PELD/MELD scores but also other parameters, such as severe complications of PHT (9). The poor efficacy of therapies for HH (serial thoracenteses or transjugular intrahepatic portosystemic shunt placement) is another element that supports the indication for LT in cirrhotic patients who develop this complication (10). In conclusion, although this is a single case, it warns physicians to always suspect HH in cirrhotic children with respiratory distress, including BA infants successfully treated with KPE, and to be aware that LT can be indicated even in case of a low PELD.

## Data Availability Statement

The original contributions presented in the study are included in the article/supplementary material, further inquiries can be directed to the corresponding author/s.

## Ethics Statement

Written informed consent was obtained from the minor(s)' legal guardian/next of kin for the publication of any potentially identifiable images or data included in this article.

## Author Contributions

All authors listed have made a substantial, direct and intellectual contribution to the work, and approved it for publication.

## Conflict of Interest

The authors declare that the research was conducted in the absence of any commercial or financial relationships that could be construed as a potential conflict of interest.

## Publisher's Note

All claims expressed in this article are solely those of the authors and do not necessarily represent those of their affiliated organizations, or those of the publisher, the editors and the reviewers. Any product that may be evaluated in this article, or claim that may be made by its manufacturer, is not guaranteed or endorsed by the publisher.
